# Population-based survey of cessation aids used by Swedish smokers

**DOI:** 10.1186/1477-7517-9-38

**Published:** 2012-12-04

**Authors:** Lars E Rutqvist

**Affiliations:** 1 Scientific Affairs Group, Swedish Match AB, Maria Skolgata 83, Stockholm 118 85, Sweden

**Keywords:** Smoking cessation, Snus, Nicotine replacement therapy, Survey, Cross-sectional, Population-based

## Abstract

**Background:**

Most smokers who quit typically do so unassisted although pharmaceutical products are increasingly used by those who want a quitting aid. Previous Scandinavian surveys indicated that many smokers stopped smoking by switching from cigarettes to smokeless tobacco in the form of snus. However, usage of various cessation aids may have changed in Sweden during recent years due to factors such as the wider availability of pharmaceutical nicotine, the public debate about the health effects of different tobacco products, excise tax increases on snus relative to cigarettes, and the widespread public misconception that nicotine is the main cause of the adverse health effects associated with tobacco use.

**Methods:**

A population-based, cross-sectional survey was done during November 2008 and September 2009 including 2,599 males and 3,409 females aged between 18 and 89 years. The sampling technique was random digit dialing. Data on tobacco habits and quit attempts were collected through structured telephone interviews.

**Results:**

The proportion of ever smokers was similar among males (47%) compared to females (44%). About two thirds of them reported having stopped smoking at the time of the survey. Among the former smokers, the proportion who reported unassisted quitting was slightly lower among males (68%) compared to females (78%). Among ever smokers who reported having made assisted quit attempts, snus was the most frequently reported cessation aid among males (22%), whereas females more frequently reported counseling (8%), or pharmaceutical nicotine (gum 8%, patch 4%). Of those who reported using snus at their latest quit attempt, 81% of males and 72% of females were successful quitters compared to about 50-60% for pharmaceutical nicotine and counseling.

**Conclusions:**

This survey confirms and extends previous reports in showing that, although most smokers who have quit did so unassisted, snus continues to be the most frequently reported cessation aid among male smokers, whereas usage of pharmaceutical nicotine was more prevalent among females. Use of snus at the latest quit attempt appeared to be associated with a higher success rate among both males and females than other reported methods, although statistically significant differences were mainly observed among males.

## Introduction

Most smokers who quit typically do so unassisted [1,2]. In many countries there is also a widespread use of various cessation aids. The efficacy of several pharmaceutical products has been documented to increase quit rates in controlled clinical trials [3,4], but their effect on a population level appears more modest in relation to the total number of excess deaths due to smoking each year [5]. In Scandinavia, population surveys have indicated that smokeless tobacco in the form of Swedish snus has been used by many smokers to quit cigarettes [6,7]. But the role of snus for smoking cessation remains controversial [8,9]. Critics have suggested that the observational character of the data on switch from cigarettes to snus precludes causal inferences. Also, until recently, there were no controlled clinical trials on the efficacy of snus for smoking cessation purposes [10,11].

Use of cessation aids may have changed in Sweden in recent years. Pharmaceutical nicotine products have become more widely available. They are sold without a prescription since 1990 and are available in convenience stores and supermarkets since 2008. The prescription drug varenicline was registered in 2006. In the public debate, some public health authorities have discouraged smokers from using snus as a cessation aid [8]. The excise tax on snus was increased substantially during the past decade. A widespread public misconception has also been documented that nicotine is the cause of much of the adverse health effects associated with smoking [12]. This may deter cigarette smokers who want to quit from using pharmaceutical nicotine products as well as snus. In the light of these circumstances, the current population-based survey was done to assess cessation strategies among Swedish smokers at their latest quit attempt.

## Methods

### Survey scheme and subject selection

The survey was based on a cross-sectional, population-based sample of subjects living in Sweden aged 18+ years. The total Swedish population in that age range is about 7.4 million. The study was designed to be geographically representative, so the number contacted in each administrative region reflected the region’s population size relative to the total population of Sweden according to Statistics Sweden. Subject selection was done in two consecutive steps. First, a population of households was defined from which selection was done by means of random digit dialing (RDD). For this purpose, the largest provider of telephone directories in Sweden (Eniro AB) supplied a database including information about private telephone extensions in Sweden. The database includes information on about 3.6 million households and is updated every three months. It does not include households with only mobile phones but does include lines registered in the Swedish Telephone Preference Service (NIX-Telefon) which allows consumers to register that they do not want to receive marketing, sales or charity collection calls by telephone. Secondly, one subject was selected from each household based on the household member having the most recent birthday. Each selected subject, if unavailable, was contacted by up to seven telephone calls, before being replaced by a new subject from another household. If unable or unwilling to participate, the selected subject was not substituted by another member of the same household.

In total, 15,178 telephone numbers were contacted which resulted in identification of 8,534 potential subjects. Of these 6,008 (70.4%) provided informed consent to participate in a telephone-based, structured interview about their tobacco habits. Of the 6,644 telephone numbers which did not result in identification of a potential subject 35% resulted in no contact, 2% were non-existent or were connected to a fax-machine, 6% concerned ineligible subjects, and 23% refused all form of contact pertinent to the survey. The 6,008 eligible subjects included in the study received no rewards or compensations for their participation. The interviews were done in November 2008 (n=1,000), and in September 2009 (n=5,008). Subject selection and telephone interviews were done by one of Sweden’s largest companies for opinion and market research (Demoskop AB) and was sponsored by Swedish Match AB.

### Telephone interview

The interview was structured through use of a questionnaire including 12 questions about present and former tobacco habits (see Appendix). For instance, ever smokers were asked about quit attempts, and use of quitting aids. All interviews were done by telephone. The interviewers used a computer aided telephone interviewing system for documentation. In total, 57 qualified interviewers were involved. Typically, each interview lasted c. 2 minutes. They were done on weekdays between 5 and 9 pm, and on weekends between 11 am and 3 pm. All interviewers worked under professional secrecy and according to the ethical guidelines published by the European Society for Opinion and Marketing Research (ESOMAR). In order to avoid the risk of bias in the data collection, the sponsor of the research was kept anonymous for both interviewers and subjects throughout the project. Also, to ensure consistency and quality of the interviews listening in and recording of interviews was done according to standard routines at Demoskop AB.

### Statistical methods

The sample size was determined on the basis of considerations related to the statistical uncertainty of estimates of the size of population subsets. Preliminary data indicated that a total sample size of c. 6,000 would generate c. 2,600 subjects with a history of smoking and quit attempts. Within a group of that size, an estimated proportion of 50% would be associated with a 95% confidence interval of +/−1.9%. For the whole survey, a proportion of 10% and 20% would be associated with a 95% confidence interval of +/−0.8%, and +/−1.0%, respectively. The statistical analyses were done by means of the statistical packages Base SAS® and SAS/Stat® (version 9.2, SAS, North Carolina, USA). Distributional comparisons were done using non-parametric (chi-squared) statistical tests. Two-sided p-values <0.05 were considered statistically significant. Reported proportions were weighted on the base of age and gender, where appropriate, so that the results would correctly represent the Swedish population according to Statistics Sweden.

## Results

### Prevalence of current tobacco use

The survey included 2,599 males and 3,409 females aged 18 through 89 years. The overall prevalence of current tobacco use (regular or occasional use) was 31.3% among males and 17.5% among females (Table 1). The prevalence of current smoking was about 15% among both males and females. Snus was used much more frequently by males (21.1%) than by females (3.8%).

**Table 1:** Prevalence of current tobacco use among the subjects included in the survey

**Males (n=2,599)**							
	Smoking:				p		
Snus use:	**Regularly**	**Occasionally**	**No**	**Total**	**Regularly**	**Occasionally**	**No**
**Regularly**	1.5	2.0	14.8	18.2	<0.001	<0.001	<0.001
**Occasionally**	0.8	0.5	1.6	2.9	0.04	0.02	0.01
**No**	7.0	3.2	68.7	78.8	<0.001	n.s.	<0.001
**Total**	9.3	5.6	85.1	100			
**Females (n=3,409)**							
	Smoking:						
Snus use:	**Regularly**	**Occasionally**	**No**	**Total**			
**Regularly**	0.2	0.3	1.8	2.3			
**Occasionally**	0.4	0.2	0.9	1.5			
**No**	10.2	3.6	82.5	96.3			
**Total**	10.8	4.0	85.5	100			

Results are presented as percentages of the total number of subjects for males and females separately weighted by age to correctly represent the Swedish population according to Statistics Sweden. P-values relate to comparisons of proportions between males and females within the same exposure category, n.s. denotes not statistically significant (p>0.05).

### Smoking history

The overall proportion of ever smokers was similar among males (47%) compared to females (44%) (Table 2). Ever smoking was generally more frequent among males aged above 54 years, and among women aged 45–64 years. Of the ever smokers, about two thirds among both males and females were former smokers, that is, they reported that they no longer smoked.

**Table 2:** Number of subjects according to age, prevalence of smoking and history/outcome of quit attempts

	**Age (years):**					
	**18-29**	**30-44**	**45-54**	**55-64**	**65+**	**Total**
**Males , N**	262	641	433	477	774	2,599
Never smoker, %	69	65	53	37	36	53
Former smoker, %	14	20	30	47	53	32
Current smoker^1^, previous quit attempts, %	10	11	11	13	6	10
Current smoker^1^, no previous quit attempts, %	7	4	5	2	5	5
**Females, N**	239	783	478	637	1,232	3,409
Never smoker, %	72	61	42	40	54	56
Former smoker, %	10	27	38	40	36	30
Current smoker^1^, previous quit attempts, %	8	9	14	14	6	10
Current smoker^1^, no previous quit attempts, %	9	3	5	6	3	5

1: Subjects reporting that they smoke regularly or occasionally,0,

Data are presented as percentages of the total number of subjects according to age and gender.

### Cessation strategies

Among former smokers, the proportion who reported unassisted quitting was higher among females (78%) than males (68%) (Table 3, p<0.001). Note that overall c. 6% of subjects reported using multiple aids so that percentages in some age groups add up to >100%. Among the subset of ever smokers who reported having made quit attempts with the use of a quitting aid (Table 4), snus was the most frequently reported method among males (63%), whereas females more frequently reported counseling (36%), or pharmaceutical nicotine (gum 35%, patch 22%). However, use of snus was substantially higher among women aged below 45 years compared to older women. In the 2009 part of the survey former smokers who reported that they had quit smoking by switching to snus (n= 254) were asked to which extent snus had helped them to quit. A total of 42% reported that snus had helped them “to a great extent” and 35% that snus had helped “to some extent”. These proportions were similar irrespective of age and gender (data not shown). To assess the success of the latest quit attempt according to reported cessation aid, we compared the number of ever smokers subdivided according to quitting method at their latest quit attempt, with the corresponding number who reported that they no longer smoked (Table 5). Of those who had used snus, 81% of males and 72% of females were successful quitters, compared to about 50-60% for pharmaceutical nicotine and counseling. There was no statistically significant difference according to gender in the success rate for the different methods. Among males, use of snus was associated with a statistically significantly higher success rate than the other specified methods (p<0.001). Among females, the difference in success rate in favor of snus was statistically significant only for the nicotine patch comparison. Analyses with exclusion of those 6% of subjects who reported using multiple aids resulted in similar estimates and conclusions.

**Table 3:** Smoking cessation method at the latest quit attempt among former smokers

	**Age (years):**					
	**18-29**	**30-44**	**45-54**	**55-64**	**65+**	**Total**
**Males, N**	34	130	131	230	409	934
Unassisted, %	54	64	62	62	75	68
Snus, %	28	28	31	28	13	22
Nicotine gum, %	5	3	4	5	4	4
Nicotine patch, %	6	0	1	3	2	2
Counseling, %	9	4	5	5	5	5
Other aid, %	8	3	2	2	3	3
**Females, N**	24	207	183	256	444	1,114
Unassisted, %	96	73	78	75	80	78
Snus, %	4	12	7	2	2	5
Nicotine gum, %	0	6	8	10	7	8
Nicotine patch, %	0	5	3	6	2	4
Counseling, %	0	7	8	10	8	8
Other aid, %	0	5	2	6	6	6

Note that some respondents reported using multiple aids which implies that percentages may add up to >100%.

**Table 4:** Reported smoking cessation aid used at the latest quit attempt

	**Age (years):**							
	**18-29**	**30-44**	**45-54**	**55-64**	**65+**	**Total**	**P**^**1**^	**P**^**2**^
**Males, N**	25	76	68	106	118	395		
Snus, %	62	68	75	61	49	63	-	<0.001
Nicotine gum, %	13	12	12	20	17	15	<0.001	<0.001
Nicotine patch, %	9	3	9	11	11	8	<0.001	<0.001
Counseling, %	20	17	21	18	22	20	<0.001	<0.001
Other, %	11	13	6	8	12	10	<0.001	<0.001
**Females, N**	7	76	74	97	121	377		
Snus, %	71	37	23	7	10	22	-	
Nicotine gum, %	15	26	43	42	36	35	0.002	
Nicotine patch, %	0	23	30	27	17	22	n.s.	
Counseling, %	0	30	43	41	39	36	0.001	
Other, %	29	24	9	23	26	22	n.s	

Data are presented as percentages of the total number of subjects according to age and gender. P^1^ relate to comparisons of proportions of subjects (all ages) using snus versus other cessation methods. P^2^ relate to comparisons of proportions between males and females (all ages), n.s. denotes not statistically significant (p>0.05). Note that some respondents reported using multiple aids which implies that percentages may add up to >100%.

**Table 5:** Outcome of latest quit attempt among subjects who reported use of a quitting aid

	**Usage (%)**	**Success rate**	**P**^**1**^	**P**^**2**^
**Males (N=1,226):**				
Snus	21.6	80.7	-	n.s.
Nicotine gum	5.2	58.1	<0.001	n.s.
Nicotine patch	2.9	54.9	<0.001	n.s.
Counseling	6.8	57.4	<0.001	n.s.
Other	3.5	70.5	n.s.	n.s.
**Females (N=1,206)**				
Snus	5.6	72.3	-	
Nicotine gum	9.0	60.7	n.s.	
Nicotine patch	5.7	48.6	0.012	
Counseling	9.3	58.9	n.s.	
Other	5.5	66.5	n.s.	

Usage refers to proportion reporting use of a specific method to the total number of subjects reporting a quit attempt. Success rate was defined as the proportion of subjects reporting no smoking at the time of the survey to the total number of subjects reporting use of the cessation aid in question. P^1^ relate to comparisons of the success rate for snus versus other methods. P^2^ relate to comparisons between males and females using the same method, n.s. denotes not statistically significant (p>0.05).

## Discussion

The current results confirm and extend previous survey data from Scandinavia in showing that, although most smokers in Sweden who have quit did so unassisted, snus continues to be the most frequently reported cessation aid among former male smokers, whereas pharmaceutical nicotine and counseling were more frequently reported by females [6,7]. However, use of snus as a cessation aid appeared to be age-related among females with higher usage among those aged below 45 years. Use of snus at the latest quit attempt was associated with a higher success rate than the other cessation aids, particularly among males, but there were no statistically significant differences in outcome with different aids according to gender. The main strengths of this survey included that it was population-based and the response rate among potentially eligible subjects was relatively high (70.4%). The final sample size of c. 6,000 was also quite large. The main weakness, like in many other observational studies, was that the collected information was based on self-reports and non-smoking status was not verified biochemically. The questionnaire was brief and did not include more detailed questions about tobacco habits, such as, smoking intensity, daily versus non-daily smoking, timing and number of quit attempts, etc. However, a more detailed questionnaire may have resulted in a decreased response rate. The random digit dialing (RDD) method could also be a limitation, since it depends on the selected subject being available and willing to answer the telephone call. However, each selected subject was contacted with up to seven calls before being replaced with a matched subject, which probably contributed to the relatively high response rate. Another possible limitation of the RDD method was that the selection of subjects was made from a database of private telephone lines which did not include mobile phones. An increasing number of individuals, particularly at younger ages, today choose to only have mobile phones in their household which means that in the future databases that do not include such phones may not be representative of the Swedish population. Although cross-sectional survey data can establish temporal relationships, such as a switch from cigarettes to snus on an individual level, causal inferences may be difficult. However, it is noteworthy that 77% of those who successfully switched from cigarettes to snus reported that snus had helped them quit smoking to “a great extent” or “to some extent”, which suggests causality.

Observations on reported quitting strategies and cessation outcomes in population surveys are probably to some extent biased by self-selection. Smokers with low nicotine dependence may more often choose to quit unassisted and succeed in remaining smokefree than smokers with high nicotine dependence. Among smokers who elect to use some form of cessation aid, self-selection between different aids is probably also a concern. Some individuals may experience oral problems with snus (possibly related to the products relatively high pH), and some simply do not favor snus for esthetic or other reasons. Such individuals are unlikely to try snus for smoking cessation purposes. Self-selection mechanisms may also be related to the long-term outcome of quit attempts. This means that one should be cautious with extrapolating conclusions based on survey data about the relative efficacy of different cessation strategies to the total population of smokers.

Relapse rates among smokers tend to be quite high up to 6–12 months after a quit attempt [13]. The current survey did not include questions about timing of the latest quit attempt. This means that the calculated “success rates” (Table 4) overestimate long term cessation outcomes as they relate in part to quit attempts that occurred only shortly before the survey. In fact, the observed “success rates”, irrespective of cessation aid, were considerably higher than biologically confirmed, long-term cessation rates typically observed in controlled clinical trials [3,4]. The first two randomized trials on the role of Swedish snus to improve quit rates among smokers were recently published [10,11]. One was conducted in Eastern Europe (Belgrade, Serbia), and the other at five sites in the U.S. Both were placebo-controlled, double-blind trials testing whether *ad lib* provision of snus affected subsequent smoking habits among adult smokers. In both studies, subjects allocated to snus were 2–3 times as likely to quit smoking completely compared to the control subjects. The trial results support that the switch from cigarettes to snus observed in population survey data is not only a temporal phenomenon but represents a true, causal association. The use of snus instead of cigarettes has been suggested to simply represent a cultural phenomenon that is particular to Scandinavia, and that snus may not perform similarly in other countries, among females, or in settings where pharmaceutical cessation aids are readily available to smokers who want to quit [14]. The results of the current survey as well as the mentioned randomized trials do not support these concerns: there was no evidence from the trials or in the survey that snus had a differential effect according to gender, there is no traditional use of oral tobacco of any kind in Serbia, and there was no evidence (mainly based on the U.S. trial results) that previous exposure to pharmaceutical nicotine modified the effect of snus.

## Appendix

Questions (response alternatives within parentheses) during the structured telephone interview (translated from Swedish).

1. Do you smoke? (Yes, regularly/ Yes, sometimes/No, never)

2. If you smoke, have you ever tried to quit but failed? (Yes/No)

3. If you don’t smoke: have you smoked in the past? (Yes/No)

4. Do you use snus? (Yes, regularly/ Yes, sometimes/No, never)

5. If you don’t use snus, have you used snus in the past? (Yes/No)

6. If you have smoked in the past, or smoke now but have tried to quit, did you start using another product when you stopped smoking, the question relates to your latest quit attempt? (Yes/No)

7. If you started using another product, which one? (snus/nicotine gum,/nicotine patch/other/don’t know)

8. If you started using snus instead of smoking, to what extent do you think snus helped you to quit smoking? (To a great extent/To some extent/To little extent/Not at all/Don’t know) (Question only included in the 2009 part of the survey)

9. If you ever smoked and ever used snus, which nicotine product did you start with? (Cigarettes/Cigars/Pipe tobacco/Snus/Nicotine gum or other pharmaceutical nicotine product/Other tobacco product/Don’t know)

10. What is your age? (−29 years/20-44 years/45-54 years/55-64 years/65+ years/Don’t want to disclose my age)

11. What is your occupation?

12. “That was all. Thank you for participating. My name is NN and I called from Demoskop” (Subject’s gender noted without asking)

## Competing interests

LER is an employee of Swedish Match AB (Sweden’s largest manufacturer of snus products).

## Authors' contributions

LER conceived of the study, was responsible for the data analysis plan, and wrote the manuscript.

## References

[B1] Yeomans K Payne KA Marton JP Merikle EP Proskorovsky I Zou KH Li Q Wilke RJ Smoking, smoking cessation and smoking relapse patterns: a web-based survey of current and former smokers in the US Int J Clin Pract 2011 65 1043 1054 10.1111/j.1742-1241.2011.02758.x 21923845

[B2] Hung WT Dunlop SM Perez D Cotter T Use and perceived helpfulness of smoking cessation methods: results from a population survey of recent quitters BMC Public Health 2011 11 592 10.1186/1471-2458-11-592 21791111 PMC3160379

[B3] Stead LF Perera R Bullen C Nicotine replacement therapy for smoking cessation Cochrane Database System 2012 11 CD000146 10.1002/14651858.CD000146.pub4 23152200

[B4] Aubin HJ Karila L Reynaud M Pharmacotherapy for smoking cessation: present and future Curr Pharm Des 2011 17 1343 1350 10.2174/138161211796150837 21524268

[B5] Apelberg BJ Onicescu G Avila-Tang E Samet JM Estimating the risks and benefits of nicotine replacement therapy for smoking cessation in the United States Am J Publ Health 2010 100 341 348 10.2105/AJPH.2008.147223 PMC2804631 20019322

[B6] Ramström LM Foulds J Role of snus in initiation and cessation of tobacco smoking in Sweden Tob Control 2006 15 210 214 10.1136/tc.2005.014969 16728752 PMC2564662

[B7] Lund KE McNeill A Scheffels J The use of snus for quitting smoking compared with medicinal products Nicotine Tob Res 2010 12 817 822 10.1093/ntr/ntq105 20622023 PMC2910876

[B8] Holm LE Fisker J Larsen BI Puska P Halldórsson M Snus does not save lives: quitting smoking does Tob Control 2009 18 250 251 10.1136/tc.2009.030221 19633141

[B9] World Health Organization, (2009). WHO Study Group on Tobacco Product Regulation Report on the scientific basis of tobacco product regulation: third report of a WHO study group 2009 Geneva, Switzerland: WHO Technical Report Series 955 20942227

[B10] Joksić G Spasojević-Tišma V Antić R Nilsson R Rutqvist LE Randomized, placebo-controlled, double-blind trial of Swedish snus for smoking reduction and cessation Harm Reduct J 2011 8 25 10.1186/1477-7517-8-25 21914165 PMC3186733

[B11] Fagerström K Rutqvist L Hughes J Snus as a smoking cessation aid: a randomized, placebo-controlled trial Nicotine Tob Res 2011 14 306 312 21994343 10.1093/ntr/ntr214

[B12] Wikmans T Ramström L Harm perception among Swedish daily smokers regarding nicotine NRT-products and Swedish snus Tobacco Induced Diseases 2010 8 9 10.1186/1617-9625-8-9 20707898 PMC2928193

[B13] Kirshenbaum AP Olsen DM Bickel WK A quantitative review of the ubiquitous relapse curve J Subst Abuse Treat 2009 36 8 17 10.1016/j.jsat.2008.04.001 18571890 PMC3151675

[B14] Scientific Committee on Emerging and Newly Identified Health Risks (SCENIHR) Health effects of smokeless tobacco products 2008 Brussels: European Commission: Health & Consumer Protection Directorate-General

